# A Novel Bioactive Emulgel with *Phlomis kurdica*: Antioxidant Potential, Enzyme Inhibition and Permeation Kinetics

**DOI:** 10.3390/gels12030240

**Published:** 2026-03-13

**Authors:** Tuğba Buse Şentürk, Timur Hakan Barak, Emre Şefik Çağlar, Emine Saldamlı, Ebru Özdemir Nath, Zafer Ömer Özdemir

**Affiliations:** 1 Department of Pharmacognosy, Faculty of Pharmacy, Acibadem Mehmet Ali Aydinlar University, 34752 İstanbul, Türkiye; tugba.avci@acibadem.edu.tr; 2 Department of Pharmacognosy, Hamidiye Faculty of Pharmacy, University of Health Sciences, 34688 İstanbul, Türkiye; 3 Department of Pharmaceutical Biotechnology, Hamidiye Faculty of Pharmacy, University of Health Sciences, 34668 İstanbul, Türkiye; s.emrecaglar@gmail.com; 4 Department of Pharmaceutical Technology, Faculty of Pharmacy, İstanbul University, 34116 İstanbul, Türkiye; eminesaldamli@istanbul.edu.tr; 5 Natural Products R&D Centre, Altınbaş University, 34144 İstanbul, Türkiye; ebru.ozdemir@altinbas.edu.tr; 6 Department of Analytical Chemistry, Hamidiye Faculty of Pharmacy, University of Health Sciences, 34688 İstanbul, Türkiye; zaferomer.ozdemir@sbu.edu.tr

**Keywords:** *Phlomis kurdica*, skin-related enzyme inhibition, LC-MS/MS, emulgel, HPLC

## Abstract

*Phlomis* L., with more than 100 species belonging to the Lamiaceae family, is a genus encompassing a diverse group of plants known for their rich phytochemical profiles and important medicinal properties. *Phlomis kurdica* Rech. fil. is a member of this genus widely distributed in the Middle East, especially in Iran, Iraq and Türkiye. In traditional medicine, *Phlomis* species have been employed in the treatment of various disorders, particularly skin conditions such as wound healing, as well as diabetes, hemorrhoids, inflammation, and gastric ulcers. The purpose of this study was to investigate the biological activities of *Phlomis kurdica* on skin-related enzymes and to evaluate its phytochemical properties using HPTLC, LC-MS/MS. Additionally, an emulgel formulation was developed with methanolic extract of the plant and characterized in terms of spreadability, textural profile analysis, pH, viscosity, and content quantification determination. In vitro release and rheology studies were carried out following the characterization investigations. According to our investigations, *P. kurdica* may be a useful component of wrinkle prevention and skin-regenerating products.

## 1. Introduction

Aging is a complex biological phenomenon characterized by progressive loss of physiological functions and changes to tissue structure [1]. Like all organs, human skin also undergoes various physiological changes with advancing age [2]. The epidermis, dermis, and hypodermis are the three layers of the skin, and each has unique physical features and purposes [3]. The dermis contains an extracellular matrix (ECM) (elastic fibers, glycosaminoglycans, and collagens) and fibroblasts, which together contribute to the durability and flexibility of the skin. Recent studies have shown that impaired ECM remodeling is associated with various pathogenic contexts, including aging or premature aging disorders [4]. Hyaluronic acid, elastin, and collagen are the primary components of the ECM and are degraded by the enzymes hyaluronidase, elastase, and collagenase, respectively [5]. Since intrinsic and extrinsic factors that contribute to aging increase the expression of these enzymes, current anti-aging strategies have focused on their inhibition. Especially, understanding the role of reactive oxygen species (ROS) in this process is crucial, since elevated ROS levels trigger the activation of skin-associated enzymes such as hyaluronidase, collagenase, and elastase, ultimately contributing to skin aging [6]. On the other hand, UV radiation, which is the biggest trigger of ROS, activates melanin and results in hyperpigmentation. The enzyme that limits the rate of melanin synthesis is tyrosinase, and its excess can also lead to a number of skin conditions such as age spots, wrinkles, sagging, and freckles. The significant amount of phenolic compounds found in plants makes them well-known antioxidants. Therefore, strong antioxidant activity plays an important role in slowing down the skin aging process and treating skin issues.

Plant extracts are a rich source of secondary metabolites with a variety of biological characteristics that can be used to replace synthetic compounds in cosmetic or medicinal products [7]. Accurate formulations contribute to increasing the therapeutic efficacy of plant extracts containing pharmacologically active secondary metabolites [8]. Emulgel is composition combining a gel and an emulsion and is growing in popularity due to its ability to effectively deliver hydrophobic medications compared to other commonly used topical methods such as creams, gels, and ointments [9]. Thixotropic, greaseless, easily spreadable, readily removable, emollient, nonstaining, long shelf life, bio-friendly, clear, and aesthetically pleasant are just a few of the benefits of using emulgels for dermatological purposes [10]. Due to these promising properties, emulgels are a great option for delivering both aqueous and oleaginous therapeutic ingredients. As a result, emulgel formulations can be used to successfully develop stable topical drug delivery systems, especially effective natural topical preparations by loading therapeutic herbal extracts [11]. Numerous prior research studies indicate that plant-based emulgels are appropriate and beneficial for the development of cosmeceutical products [12].

The genus *Phlomis* (family Lamiaceae) comprises over 100 species native to the Mediterranean region, with a distribution range extending from Central Asia to China, with 34 species in Türkiye [13,14]. Traditional preparations derived from the aerial parts of *Phlomis* are commonly employed in Anatolia for the promotion of wound healing and the management of inflammatory conditions [15]. Recent studies have also reported that various *Phlomis* species exhibit antioxidant, antimicrobial, anticancer, anticholinesterase, antiparasitic and antimutagenic activities [16,17,18,19,20]. Findings on the genus *Phlomis* indicate the need for further studies to obtain more detailed information regarding the chemical profiles and bioactivities of these species.

The aim of this study was to determine the inhibitory capacity of *Phlomis kurdica* (PK) on skin-related enzymes, its antioxidant activity and to elucidate its phytochemical profile. Finding inhibitors of elastase, collagenase, tyrosinase, and hyaluronidase enzymes, as well as novel agents with high antioxidant potential, could help prevent sagging, hyperpigmentation, and loss of skin elasticity. In addition, emulgel formulation was developed from the main methanol extract and characterized. The promise of a number of natural extracts derived from plants to treat skin conditions has been documented in the literature; however, for a variety of reasons, the majority of these extracts have not been developed into innovative drug delivery methods. In light of the growing demand for products derived from natural sources, this study focuses on developing and evaluation of plant-based emulsion gel formulations. The current investigation is mainly positioned within the framework of dermocosmetic (cosmeceutical) development. The formulation results given below present support for the possible addition of PK to topical formulations designed to reduce hyperpigmentation and photoaging symptoms.

## 2. Results and Discussion

### 2.1. Phytochemical Evaluation

The broad-spectrum therapeutic properties of plants are generally attributed to the abundance of bioactive secondary metabolites found in plant species. Consequently, clarifying the phytochemical profiles of plants using sophisticated chromatographic analysis discloses the amounts and types of components in plant extracts, thereby facilitating the search for bioactive substances that may aid in the development of effective pharmaceuticals [21].

**Figure 1:**
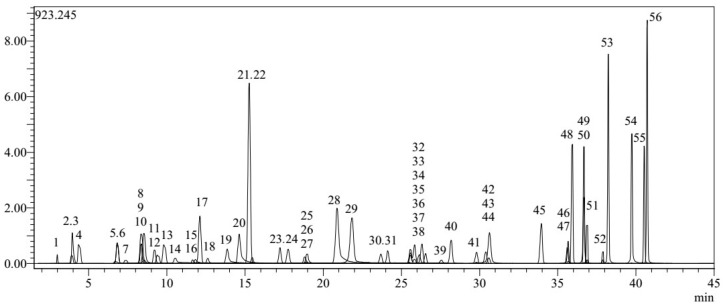
LC-MS/MS chromatograms of the PKM.

First, the present study used LC-MS/MS to qualitatively and quantitatively assess 53 phytochemicals in the main methanol extract of the PK. (PKM). The results are given in Table 1 and Figure 1.

**Figure 2:**
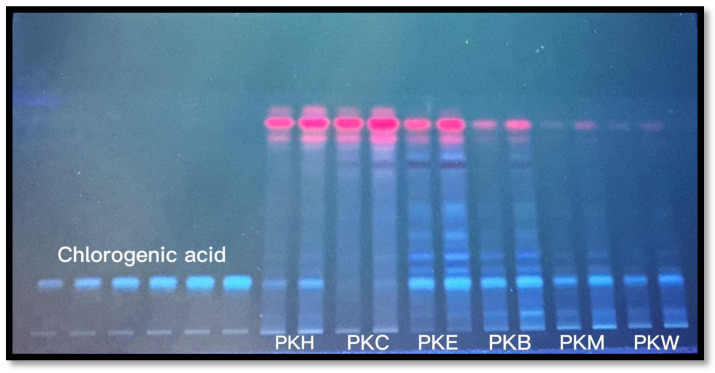
HPTLC chromatograms of: PKH: hexane fraction of PK, PKC: chloroform fraction of PK, PKE: ethyl acetate fraction of PK, PKB: n-butanol fraction of PK, PKM: methanolic extract of PK, PKW: remaining water fraction of PK. Mobile phase: toluene: ethyl acetate: formic acid (7:5:0.5). Derivatization: NPR reagent. Visualization: 366 nm.

In present study, quinic acid was the most abundant phytochemical in PKM extract, detected by LC-MS/MS, with 85.396 μg/g. It was followed by chlorogenic acid with 20.379 μg/g and luteolin 7-*O*-glucoside with 5.373 μg/g. Similar to our findings, a previous study by Yagi et al. reported the detection of 93 chemical compounds, including chlorogenic acid, quinic acid and luteolin-7-*O*-glucoside, in the methanol extract of PK [22]. Earlier investigations that have been published have also revealed a variety of flavonoids and phenylpropanoids in the plant, such as leukoseptoside A and B, verbascoside, alissonoside, and forcytoside B [23,24]. In the HPLC analysis of the methanol extract prepared from the aerial parts of PK collected from Iran in 2023, chlorogenic acid was identified as the dominant phenolic compound [25]. In a study conducted by Saraçoğlu et al. on another *Phlomis* species, *Phlomis lycia*, chlorogenic acid was isolated from the plant [26]. Izol reported that 19 chemicals were detected in *Phlomis capitata*; quinic acid (4.883 mg g^−1^) was the most abundant, followed by chlorogenic acid (4.36 mg g^−1^) [27]. According to another investigation, chlorogenic acid is the most prevalent acid in *P. armeniaca*, followed by salicylic acid, 4-dihydroxybenzoic acid, and quinic acid [28]. These data indicate that chlorogenic acid is widely present in species of the genus *Phlomis* and is an important component of the phytochemical profile. The objective was to ascertain the distributional discrepancies among the fractions of chlorogenic acid identified in the primary methanol extracts via HPTLC and analyzed using LC-MS/MS. The outcomes of HPTLC are presented in Table 2 and Figure 2.

Chlorogenic acid was detected in all fractions of PK, with the highest amount measured in the ethyl acetate fraction (205 ± 16.73 μg/mg). This was followed by the n-butanol fraction (125.97 ± 7.09 μg/mg) and the methanol extract (56.88 ± 0.89 μg/mg), respectively. The solubility of the compound in moderately polar solvents is particularly highlighted by the decreased levels in hexane, chloroform, and aqueous fractions; this is consistent with its phenolic structure and regular extraction behavior in phytochemical investigations.

### 2.2. Formulation and Characterization of Emulgel

Currently, consumers are progressively drawn to skincare products and cosmetics formulated with natural elements. This trend has led numerous cosmetic manufacturers to launch more products with plant extracts recognized for its anti-aging, anti-wrinkle, and skin-brightening attributes, among other advantages [29]. These plant-based ingredients may treat a variety of skin conditions and are frequently utilized in topical skincare treatments. However, the majority of these assertions are not supported by science [30]. Consequently, the cosmetics industry has made it a priority to develop herbal-based formulations with benefits that have been scientifically verified.

Since the skin is the most accessible organ, topical application is the best way to administer active substances for skin-related treatments. Targeted application, and avoiding gastrointestinal problems and metabolic difficulties associated with oral administration are just a few benefits of topical medication delivery. Even though this approach has been used for a while, new methods and tools are always being created to improve patient compliance [31]. As a result of the growing interest in plant-based phytochemicals, emulgel systems that use natural plant extracts have drawn attention as an innovative and successful technique in cosmetology [32]. Emulgel formulations containing different herbal extracts have demonstrated encouraging benefits in earlier research [32,33,34].

PKM extracts were used in this investigation to create emulgel formulations. The findings of the in vitro characterization, including measurements of pH and viscosity, are shown in Table 3.

The pH of F (5.40 ± 0.10) corresponds to the generally advised moderately acidic range for topical formulations, promoting barrier compatibility and minimizing irritation risk. PKF (6.03 ± 0.01) was somewhat elevated; however, it remained within the upper limit of commonly referenced ranges for topical treatments; such minor fluctuations may be mitigated by the skin and could be pertinent based on the intended indication and skin condition (healthy versus irritated skin). The minimal fluctuation noted for PKF pH signifies excellent consistency of this parameter [35,36,37].

The viscosity of F was higher than that of PKF (24.023 ± 0.228 P vs. 19.027 ± 0.206 P), indicating that the extract’s integration made the gel less resistant to flow. Carbopol-based gels exhibit sensitivity to formulation composition, including cosolvents, electrolytes, and pH/neutralization state, all of which can influence polymer swelling and network formation, thus affecting viscosity and application efficacy [38,39]. A moderate reduction in viscosity can enhance spreadability and application ease; however, excessively low viscosity may diminish residence time or lead to runoff. Consequently, supplementary assessments, including spreadability, extrudability, and short-term stability across varying temperatures, are generally employed to validate product efficacy [40].

PKF demonstrated a drug/extract content of 101.069 ± 5.375%, aligning with adequate incorporation and near-target test recovery in semisolid topical formulations. Content homogeneity is crucial as it indicates proper dispersion of the active ingredient within the vehicle and is directly associated with consistent dosage during patient administration. The observed variability indicates that further mixing, process modification, or a review of the sampling technique (e.g., numerous sampling locations within the batch) could enhance uniformity, as is typically assessed for semisolid dosage forms.

### 2.3. Texture Profile and Spreadability Analysis

In order to evaluate the spreadability and texture profiles of these formulations, extensive research was conducted. According to the results of the spreadability inquiry and the texture profile analysis of the emulgel formulation, the findings are presented in Table 4 and Table 5.

The order in which the components of a formulation are placed and the interactions that occur between them were better understood by looking at the mechanical textural characteristics of a semisolid emulsion system. TPA finds these mechanical properties by applying an external compressive force to the sample and assessing the model’s ability to produce both reversible and irreversible deformations [41,42,43]. The TPA methodology is a quick and simple means to learn about the physical structure of an emulsion system [44]. At a temperature of 25 °C, the formulations’ mechanical and textural qualities were evaluated. It is the initial maximum compressive force that is responsible for determining the hardness (N or g) of a product. Hardness is defined as a material’s resistance to deformation. The hardness of a gel, which persists after application to the skin, indicates the ease of application on the patient’s skin. The tackiness value, associated with sticky properties, determines the ease of probe removal from the formulation. An increased level of tack, signifying enhanced tissue surface adherence, is a desirable characteristic for prolonged drug retention as it facilitates drug retention. A negative force field is employed to determine the adhesiveness of the gel surface during the initial compression cycle. This transpires when an attractive force exists between the probe and the gel surface. The gel sample was deformed due to initial compression, and the springiness or elasticity of the gel during its recovery after deformation characterizes its regeneration. The viscosity of the formulations serves as a measure of their reaction to repeated shear stresses. The viscosity of the formulation indicates its response to repeated shear stresses. The degree of stickiness of a product can be assessed by comparing its capacity to reorganize after one deformation with its capacity to reorganize after a different deformation. Upon analyzing the formulations, it was found that those containing extracts exhibited reduced hardness, adhesiveness, and elasticity compared to the blank formulations. The viscosity data are also consistent with these results. The influence of ambient ion concentrations on the gel-forming chemical employed is the primary cause for the observed results. The methanol content of the extracts was found to adversely affect the gelation of the drug. The mechanical qualities of the antibacterial gel loaded with plant extract, developed by Zamaora and his colleagues, were determined to be inferior to those of the control gel [45]. The reduced mechanical properties of both the extract-loaded gel and the blank gel are significant for the patient. The mechanical qualities of the blank gel are superior. Due to its low viscosity and rapid gelling properties, it produced a gel that was easy to handle. Similarly, it is now feasible to eliminate any adverse effects on the skin with increased assurance. In topical formulations, spreadability is a critical factor since it significantly affects patient compliance. A formulation with excellent spreadability facilitates administration and enhances skin coverage, hence augmenting the therapeutic efficacy of the product [46]. The spreadability test assesses the variation in force over time, indicating attributes such as firmness, cohesion, spreading work (or shear work), and extrusion force (or adhesion work). The strength of the formulation can be assessed by measuring its hardness, defined as the maximum positive force required to induce deformation. Increased hardness indicates a more potent composition. The spreadability of the formulation is determined by quantifying the shear effort, represented by the area under the positive force curve. The resistance to adhesion or flow is depicted by the negative segment of the force graph in the method. This segment is affected by the mass of the sample positioned on the rotating probe. The cohesive force necessary to detach the gel from its container is indicated by the greatest negative value, while the region beneath the negative curve signifies the cohesive effort required for this separation [47]. The relationship between spreadability and hardness and shear work is inversely proportional [48]. There was a correlation between the lower gelation capacity and the mechanical properties of extract-loaded gels, which influenced the spreadability outcomes. In accordance with the values obtained from the viscosity and TPA, the experimental findings revealed that the gels loaded with extract exhibited greater spreadability in comparison with the formulations that were left completely blank.

### 2.4. Rheology Studies

The rheological evaluation of the examined formulations revealed detailed information about the behavior of both the carrier gel matrix and the extract-loaded gel system, particularly in terms of flow characteristics, viscoelastic responses, and structural stability under changing conditions. At 25 °C and 32 °C, the empty formulation had a higher storage modulus (G′) than loss modulus (G″), indicating gel-like characteristics over the temperature range. At lower frequencies, the difference between G′ and G″ rapidly narrowed, indicating a weak gel nature. The viscoelastic equilibrium altered with increasing deformation time scales.

The loss tangent (tan δ = G″/G′), which expresses the ratio of dissipated to stored deformation energy, is a critical parameter for evaluating the balance between the viscous and elastic components of gel systems. Across all formulations examined, the elastic modulus (G′) consistently exceeded the viscous modulus (G″), resulting in tan δ values lower than 1. This behavior indicates that the gels predominantly exhibit elastic, solid-like characteristics within the low-strain region, in agreement with previous findings reported by Dabbaghi et al. [49]

Such elastic dominance is particularly desirable for maintaining structural integrity under small deformations during application.

**Figure 3:**
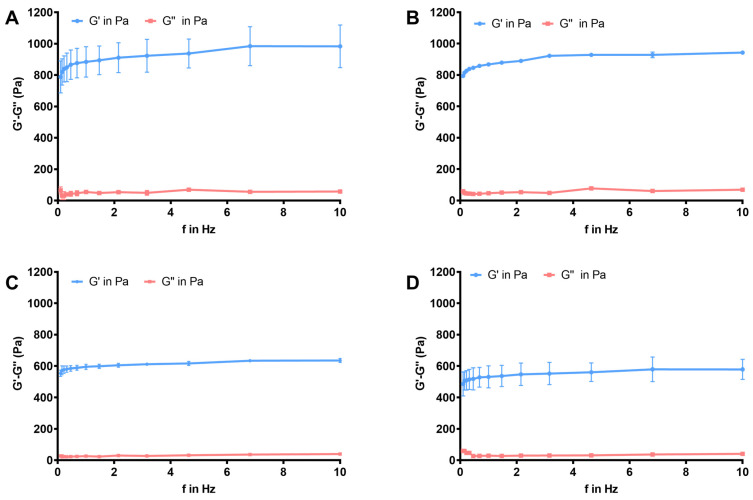
Frequency-dependent changes in viscoelastic properties of the formulations (n = 3). (**A**) F 25 °C, (**B**) F at 32 °C, (**C**) PKF at 25 °C, (**D**) PKF at 32 °C.

In the extract-loaded gels, a marked difference between G′ and G″ was observed at 25 °C, suggesting a well-developed elastic network at room temperature. Although this difference diminished when the temperature was increased to 32 °C, the condition G′ > G″ was preserved and tan δ values remained below unity (Figure 3, Table 6). This indicates that the incorporation of the PKM did not compromise the fundamental viscoelastic structure of the gel, even under conditions mimicking physiological temperature. The retention of elastic dominance with increasing temperature suggests that the gel network remains sufficiently interconnected and resistant to thermal softening.

Interestingly, despite the overall stability of the viscoelastic moduli, the extract-loaded gel exhibited a considerable increase in viscosity at 32 °C at a low frequency (0.1 Hz) compared to 25 °C. This temperature-dependent viscosity enhancement was substantially less pronounced in the blank gel, highlighting the role of the PKM in modulating the rheological response of the system. The observed behavior implies that extract incorporation may promote additional intermolecular interactions or structural rearrangements that become more prominent at elevated temperatures, leading to a heat-responsive or thermo-sensitive gel-like behavior.

From a practical perspective, this thermally induced increase in viscosity is highly advantageous for topical or localized delivery systems, as gels that become more viscous at higher temperatures can exhibit prolonged residence time at the site of application.

This study provides important information about the viscoelastic behavior of blank and PKM-loaded gels at different temperatures and oscillation frequencies. The fact that tan δ values remained well below 1 in all formulations and across all tested frequency ranges clearly demonstrates that the systems predominantly exhibit elastic (solid-like) characteristics. This indicates that the gel network structure maintains its integrity against low- and high-frequency deformations, and that elastic recovery is more prominent than viscous flow. For the blank gel, tan δ values at 25 and 32 °C generally remained low and stable as the frequency increased. The absence of a significant increase in tan δ with increasing temperature suggests that the viscoelastic equilibrium of the blank gel is not affected by temperature changes and that the gel network exhibits a thermally stable structure. The PKM-loaded gel, however, showed a more pronounced behavioral change with temperature and frequency variations. At 25 °C, as with the blank gels, low tan δ values were observed across all frequencies, indicating that PKM loading did not alter the elastic behavior of the gel system. However, when the temperature is increased to 32 °C, the increase in the tan δ value, especially at 0.1 Hz compared to the blank gel at 25 °C, indicates that the presence of PKM in low-frequency (long-time scale) deformations increases viscosity. Despite this, the fact that tan δ remains below 1 shows that the system retains its elastic-dominant character. The dynamic viscosity (η*) results strongly support this interpretation.

**Figure 4:**
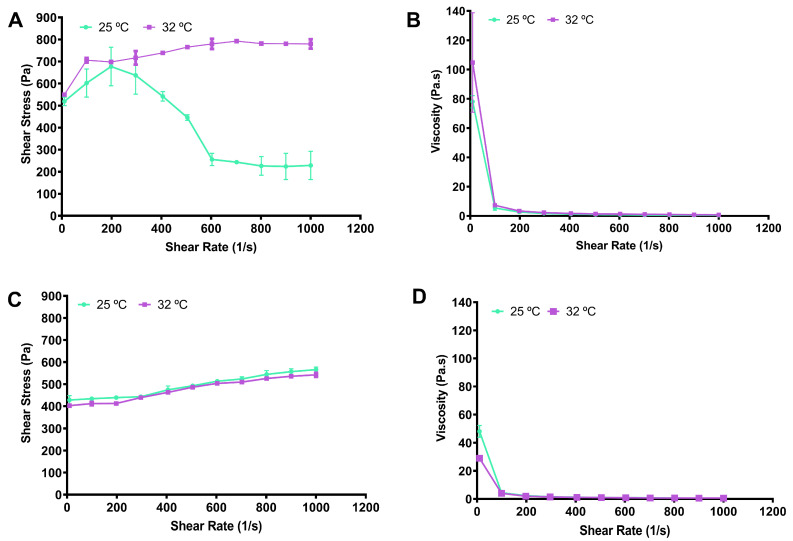
Flow curves of (**A**) F at 25 °C and at 32 °C, (**C**) PKF at 25 °C and at 32 °C, and viscosity curves of (**B**) F at 25 °C and at 32 °C, and (**D**) PKF at 25 °C and at 32 °C.

The significant decrease in η* with increasing frequency in both formulations indicates that the gels exhibit shear-thinning (pseudoplastic) behavior, a property highly advantageous for topical and local applications (Figure 4). Pseudoplastic behavior becomes more pronounced as cross-linking and interaction between molecules in different carriers increase [50]. While the temperature increase has a limited effect on η* in the blank gel, the significant viscosity increase observed at 32 °C at 0.1 Hz in the PKM-loaded gel points to a structural rearrangement with increasing temperature. This finding clearly demonstrates that the system acquires a heat-responsive character after PKM loading. The higher viscosity exhibited by the PKM-loaded gel at physiological temperatures suggests that post-application gel flow can be limited, thus extending the residence time. Overall, these results reveal that PKM loading not only maintains the viscoelastic equilibrium of the gel system but also imparts a temperature-sensitive, functional rheological response. The combined evaluation of Tan δ and η* data shows that the PKM-loaded gel exhibits a controlled viscosity increase at physiological temperatures while maintaining its elastic-dominant structure, thus offering optimized performance for target applications.

### 2.5. In Vitro Release Studies

**Figure 5:**
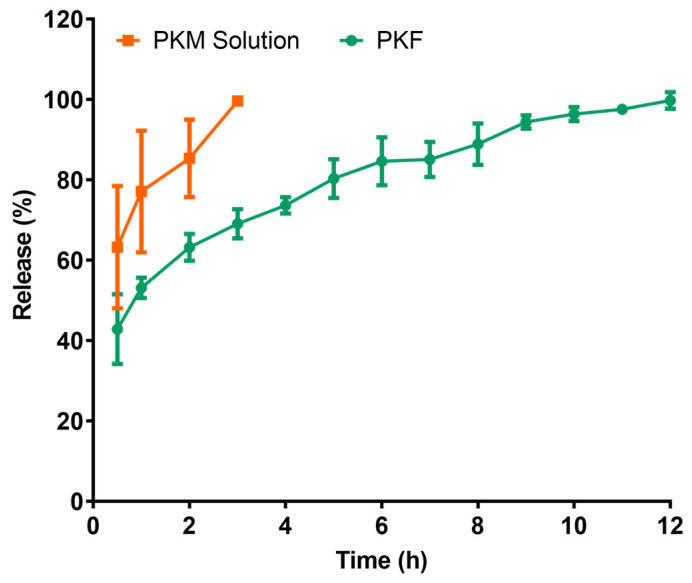
In vitro release studies of PKF and the PKM Solution.

In vitro release investigations of PKM extract loaded gel and the chlorogenic acid solution were performed using dialysis membranes. Quantification of chlorogenic acid in the release media was carried out by HPLC analysis of aliquots withdrawn at predetermined time intervals. The cumulative release profiles of both formulations are presented in Figure 5**.** The overall release percentages were determined to be 99.620 ± 0.976% for the chlorogenic acid solution at 3 h and 99.736 ± 2.062% for the PKM extract-laden gel after 12 h.

#### 2.5.1. Kinetic Release Modeling

The kinetic modeling of in vitro release studies presents a comparative kinetic evaluation of in vitro release data obtained from PKM loaded gel and the chlorogenic acid solution. The present investigation compares the kinetic evaluation of PKM-containing gel and chlorogenic acid data utilizing the zero-order, first-order, Higuchi, Hixson–Crowell, and Korsmeyer–Peppas models in in vitro release tests. The key findings are given in Table 7. The gel formulation showed a regulated release profile with progressive drug release. The Korsmeyer–Peppas (R^2^ ≈ 0.996), Higuchi (R^2^ ≈ 0.989), and zero-order (R^2^ ≈ 0.922) kinetic models provided the most accurate fit. The first-order kinetics (R^2^ ≈ 0.850) and Hixson–Crowell (R^2^ ≈ 0.876) models exhibited moderate correlation. Within the first few hours, the solution form demonstrated extremely quick and nearly total release. The Korsmeyer–Peppas model provides valuable insights into release mechanisms by capturing both diffusion-driven processes and matrix-related factors such as swelling and erosion [51]. The release exponent (n ≈ 0.280) derived from the Korsmeyer–Peppas equation implies that the release of chlorogenic acid from this gel formulation Fickian diffusion (n < 0.5) and demonstrates a controlled swelling mechanism. This indicates that the systems have high potential for mechanical robustness and controlled release capability [52].

#### 2.5.2. Similarity Studies

The model-independent approach that Moore and Flanner presented was utilized in order to carry out the comparative statistical analysis of the in vitro release patterns. This technique makes use of two indices, namely the difference factor (*f*_1_) and the similarity factor (*f*_2_), in order to quantify the degree of similarity that exists between dissolution curves. According to the criteria that have been developed, *f*_1_ values that are lower than or equal to 15 and *f*_2_ values that fall within the range of 50 to 100 indicate that the two release profiles can be deemed to be comparable [53]. In order to make comparisons within the formulation, statistical analysis was performed.

The results of the calculation of the difference and similarity factor for pair-wise intraformulation comparisons are presented in Table 8. When compared to PKS, the release profile of PKF was statistically shown to be considerably different (*f*_1_: 23, *f*_2_: 33). Furthermore, when compared to PKF, the release profile of PKS was shown to be significantly different (*f*_1_: 30, *f*_2_: 33).

The fact that the results of formulations f1 and f2 are statistically different suggests that the release mechanisms and profiles of the two formulations are distinct from one another. It is important to note that these two approaches to medicine delivery are distinct from one another and are intended to accomplish different therapeutic goals. The gel formulation performs gradually and consistently, in contrast to the solution, which operates quickly. Further validation of the controlled-release properties of PKF is provided by these findings, which also provide support to the possibility of its employment in circumstances that call for extended released bioactive substances.

### 2.6. In Vitro Antioxidant Tests

Although cellular metabolism normally generates ROS, oxidative stress is a pathological state that occurs when the balance of free radicals to antioxidants changes in preference for free radicals [54]. Particularly when exposed to UV light, skin cells produce and accumulate ROS quickly, which can result in photoaging. ROS-induced photo-oxidative stress is believed to be the primary pathogenic process that damages ECM proteins, which cause wrinkles, and causes photomutagenesis, which causes skin cells to develop cancer [55]. Furthermore, ROS can lead to hyperpigmentation problems in the skin by triggering the production of α-melanocyte-stimulating hormone (α-MSH) in keratinocytes, initiating the activation of the tyrosinase enzyme, and stimulating melanin synthesis in melanocytes [56]. As a result, antioxidant compounds that help scavenging ROS are extremely beneficial resources in treating skin conditions like wrinkles, blemishes, and aging [57].

Plants are well-known antioxidants due to their high content of phenolic compounds [58]. The main reason of phenolic compounds’ antioxidant effects is their redox characteristics, which can be crucial for adsorbing and neutralizing free radicals, quenching singlet and triplet oxygen, and breaking down peroxides [59]. In this study, antioxidant activity was evaluated using CUPRAC, FRAP, TOAC, and DPPH radical scavenging activities in PKM and all its subfractions. The results are given Table 9.

In PK, the highest antioxidant potential was detected in the PKE and PKB fractions. These fractions were also the richest in chlorogenic acid, a phenolic acid. Previous phytochemical studies of the *Phlomis* genus have shown that they contain iridoids, flavonoids, phenylpropanoids, phenylethanoids, lignans, neolignans, diterpenoids, alkaloids, and essential oils [60]. Some of the biological effects of this genus have been associated with the presence of these flavonoids and other phenolic compounds. In a study by Zhang et al. [61], the antioxidant activities of acetone and methanol extracts obtained from the leaves of *Phlomis umbrosa* Turcz. and *Phlomis megalantha* Diels were investigated. Among the different extracts, the acetone extract of *P. megalantha* exhibited the highest antioxidant activity. The predominant phenolic compound in this plant was chlorogenic acid (26.15 ± 0.20 μg/g extract), followed by rutin (13.92 ± 0.29 μg/g extract), rosmarinic acid (7.78 ± 0.42 μg/g extract), and benzoic acid (6.00 ± 0.25 μg/g extract) [61]. In another study, the antioxidant activities of methanol (MEPT) and water (WEPT) extracts taken from the aerial parts of *P. tuberosa* L. were compared. In addition, total phenol and flavonoid contents were also analyzed. Chlorogenic acid was determined as the predominant phenolic compound in MEPT, and its amount was eight times higher (7671.48 ng/mL) than in the water extract. In addition, the methanol extract contained higher amounts of phenolic and flavonoid substances compared to the water extract. The results consistently showed that the methanol extract had higher antioxidant activity than the water extract in all experiments [60]. *Phlomis* species are rich in caffeoyl quinic acids, which include substances such as chlorogenic acid, and have been associated with antioxidant activity. Chlorogenic acid is an ester formed between caffeic acid and quinic acid, and its well-known antioxidant function has been widely reported [62]. In an earlier investigation by Wu, 70% ethanol, methanol, and water extracts of Flos Lonicerae were produced, and their antioxidant capabilities were assessed using DPPH and FRAP tests to determine whether chlorogenic acid significantly contributes to antioxidant activity. Higher levels of chlorogenic acid were shown to be more effective at scavenging DPPH radicals and converting Fe^+3^ to Fe^+2^. It was discovered that the water extract had more than 50% less chlorogenic acid than the ethanolic and methanolic extracts. In addition, the antioxidant capacity of chlorogenic acid purified from 70% ethanol extract was compared with that of ethanol extract and ascorbic acid. Purified chlorogenic acid exhibited a much stronger antioxidant capacity than both the extract and ascorbic acid. The results showed that Flos Lonicerae extracts have antioxidant activity related to total phenolic compound content and mainly contributed through the presence of chlorogenic acid [63]. In conclusion, it can be claimed that chlorogenic acid plays an important role in antioxidant activity and may be beneficial in the prevention of some oxidative diseases.

### 2.7. Inhibition Potential on Skin-Related Enzymes

The structure and function of the skin can be altered by a variety of chemical and physical factors [64]. Specifically, the evolution of photoaging results from a series of responses triggered by the skin’s exposure to UV light. Long-term UV radiation exposure can cause sunburn (erythema), wrinkles, skin pigmentation changes including melasma, immune system suppression, inflammation, skin cancer, and a reduced capacity to heal wounds [65].

**Figure 6:**
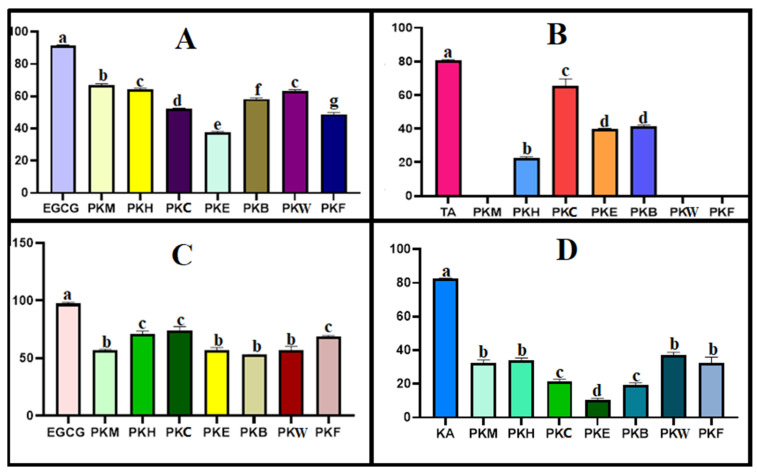
Skin-related enzyme inhibitory effect of PK, novel formulation and all fractions. Different letters indicate significance (*p* < 0.05). Inhibitory effects of PKM (methanolic extract of PK 1 mg/1 mL), PKH (hexane fraction of PK 1 mg/1 mL), PKC (chloroform fraction of PK 1 mg/1 mL), PKE (ethyl acetate fraction of PK 1 mg/1 mL), PKB (n-butanol fraction of PK 1 mg/1 mL), PKW (remaining water fraction of PK 1 mg/1 mL), PKF (emulgel formulation of PK) on elastase (**A**), hyaluronidase, (**B**) collagenase (**C**), tyrosinase (**D**). Standards: EGCG: epigalloctechin gallate (1 mg/mL for anticollagenase activity, 0.25 mg/mL for antielastase activity), TA: tannic acid (1.1 mg/mL), KA: kojic acid (1 mg/1 mL).

Fibroblasts and significant structural proteins including collagen and elastin make up the complex structure that is the extracellular matrix (ECM) of the skin. Collagenase is in responsible of the extracellular matrix’s (ECM) remodeling, which includes the breakdown of collagen. The degradation of elastin in the extracellular matrix is mainly caused by the serine proteinase elastase. The enzyme hyaluronidase degrades hyaluronic acid. The synthesis of these enzymes is increased by prolonged exposure to UV light, which causes wrinkles and photoaging of the skin [66]. Moreover, premature aging or photoaging is closely linked to changes in skin pigmentation. Tyrosinase is the enzyme that catalyzes the production of melanin in melanocytes, and melanin is the primary pigment responsible for the color of the skin. The potential cosmetic applications of natural compounds with the production of melanin inhibitory activity, such as skin-whitening or antibrowning treatments, are of interest [67]. In vitro studies offer numerous benefits for getting reliable information on the biological effects of substances. The current investigation examined the PK extracts in vitro inhibitory effects on the enzymes hyaluronidase, collagenase, elastase, and tyrosinase; the findings are shown in Figure 6.

PKM extract was found to contain a high amount of quinic acid, 85.396 μg/g. Regarding antityrosinase activity, PKM was found to have a lower inhibitory capacity compared to the known inhibitor kojic acid, but this capacity did not create a statistically significant difference with the best-performing fractions. Previous studies on antityrosinase activity in different *Phlomis* species showed similar low activity results compared to kojic acid. In a study on *P. rigida*, the IC_50_ value of the extract (1.092 mg/mL) was found to be much higher than that of kojic acid (0.049 mg/mL), meaning its efficacy was reported to be lower [68]. Similarly, in a study on three different *Phlomis* species (*P. nissolii*, *P. samia*, *P. sieheana*), dichloromethane and methanol extracts obtained from all plants showed antityrosinase activity, but their % efficacy was all found to be lower than that of kojic acid [69]. In HPTLC, the chlorogenic acid content of the fractions for PK was compared. The highest chlorogenic acid content was detected in the PKE fraction; however this fraction showed the lowest antityrosinase activity. Consistent with our findings, in a previous study where the antityrosinase activity of ethyl acetate, methanol, and water extracts of *P. armeniaca* was measured, although the highest chlorogenic acid content was detected in the methanol extract (11.95 mg/g extract), no inhibitory activity was observed in this extract [70]. Although the methanol extract of *P. nissolii* contained 9.18 ± 0.713 mg/g extract and the ethyl acetate extract contained 1.53 ± 0.060 mg/g extract of chlorogenic acid, the ethyl acetate extract showed higher tyrosinase enzyme inhibition [71]. A direct correlation between chlorogenic acid content and anti-enzyme activity has not been established. Therefore, enzyme inhibition may instead be attributed to the combined or synergistic effects of multiple phytochemicals present in the extracts. Such multi-component interactions are common in plant-derived extracts and may lead to biological activities that cannot be explained by a single compound alone.

## 3. Conclusions

The standardization of formulations made from plants is becoming increasingly common worldwide due to their long history of use and low side effect profile. This study was designed to investigate the phytochemical profile, antioxidant capacity and potential for inhibiting essential skin-related enzymes of PK due to gaps in the literature and the need for comprehensive research to explore the beneficial health potential of PK. Furthermore, an emulgel formulation was created using the primary methanolic extract (PKM) of the plant and evaluated for spreadability, viscosity, pH, textural profile analysis, and content measurement. After the characterization investigations, in vitro release and rheology studies were conducted. The findings demonstrated antioxidant potential and enzyme-related inhibitory effects of PK. After further investigation, PK-loaded emulgels may represent a promising topical formulation with antioxidant and enzyme inhibitory potential, provided that their efficacy and safety are confirmed through advanced in vitro and in vivo studies.

## 4. Materials and Methods

### 4.1. Chemicals

All chemicals, reagents, enzymes and references were analytical grade or purer.

### 4.2. Plant Material

In 2021, PK was gathered from Venk Village in Centre, MALATYA. Dr. Ebru ÖZDEMİR NATH provided a botanical description of plant samples. The plant’s voucher specimen was stored at Altınbaş University’s Pharmacy Herbarium in Istanbul, Turkey (HERA 263).

### 4.3. Preparation of the Extract

Air-dried plant materials were ground into a fine powder prior to extraction. The ingredients were then allowed to macerate for three days at room temperature in a dark environment using a 4:1 methanol/water combination (four hours in an orbital shaker). After the macerate was filtered through double-layer filter paper twice, the filtrates were mixed, put in tared balloons, and allowed to evaporate in a rotavapor until they were completely dry. After that, it was lyophilized to extract the water and frozen at −80 °C. %5.05 was determined to be the total extraction yield. The extracts were then combined with enough water and put through liquid–liquid extraction in the separating funnel using solvents ranging in polarity from the most non-polar to the polar. Yield calculations were finished and stored at −20 °C after the solvents of the resulting fractions were evaporated in a rotavapor. Ultimately, methanol, n-hexane, chloroform, ethyl acetate, n-butanol, and residual water were extracted from the plant along with the primary extract and five subfractions.

### 4.4. Phytochemical Investigation

#### 4.4.1. Determination of Phenolic Compounds by LC-MS/MS

A method earlier developed by Yılmaz [72] was used to identify and quantify 53 phytochemical constituents in the methanolic extract of PK using LC-MS/MS. Tandem MS equipment and a Nexera type UHPLC (Shimadzu, Kyoto, Japan) were used for the quantitative analysis. Shimadzu Lab Solutions Insight software was used to process the data from the LC-ESI-MS/MS analysis.

#### 4.4.2. Quantification of Marker Bioactive Compounds by HPTLC

Chlorogenic acid was measured both quantitatively and qualitatively using an HPTLC system in accordance with the previously employed technique [73]. HPTLC silica gel 60 F 254 plates measuring 20 cm by 10 cm were utilized for this. The mixture of EtOAc-CHCI_3_-FA-AA-H_2_O with a volume of 100:25:10:10:11 was identified as the mobile phase. A 0.45 µm syringe filter was used to filter each sample solution, which was generated at 1 mg/mL and standards at 100 µg/mL in methanol. The CAMAG Automatic TLC Sampler IV (CAMAG, Muttenz, Switzerland) was used to apply 6 mm bands of extracts (2–40 µL) and standards (2–12 µL) on the silica plates. Development took place in the CAMAG Automatic Development Chamber-2 (ADC-2) (CAMAG, Muttenz, Switzerland), which was pre-conditioned and saturated with MgCl_2_ for ten minutes at 33% relative humidity. Densitometric analysis was performed using a CAMAG TLC Scanner IV (CAMAG, Muttenz, Switzerland) in fluorescence mode with a slit size of 5 × 0.2 mm and a scanning speed of 20 mm/s following derivatization with Natural Product Reagent (NPR). By comparing the area under the curve (AUC) at 366 nm to a calibration curve, the standard content was ascertained. Device control and analysis were performed using VisionCATS 4.2 software, with correlation coefficients (r^2^) greater than 0.98.

### 4.5. Development of Emulgel Formulation

Emulgel formulations were selected as the vehicles for the principal methanol extracts of the plants. The prior formulation served as a carrier system [74]. The formulation consisted of two separate phases, namely an oil phase and an aqueous phase. The oil phase was prepared to contain 5% oleic acid, 1.37% Span 80, and 0.1% methyl paraben as a preservative. The aqueous phase comprised 1.8% glycerin, 3% ethyl alcohol, 3.63% Tween 60, and 0.1‰ propyl paraben as a preservative. Both phases were heated separately to 60 °C, after which the aqueous phase was added dropwise to the oil phase under continuous stirring. Following emulsion formation, Carbopol 934 was incorporated into the system at a concentration of 2% and stirring was continued until complete hydration. Subsequently, a few drops of triethanolamine were added to induce gelation of the formulation.

The same procedure was applied for the preparation of the plant extract-loaded formulation. The plant extract was first dissolved in the oil phase at 4 °C, after which the emulsification and gelation steps were carried out as described above. To enhance the stability of the emulsion, the hydrophilic–lipophilic balance (HLB) was adjusted to between 11 and 12.

#### 4.5.1. Characterization Studies of the Formulation

##### pH Measurement

The formulations’ pH was measured using a pH meter Mettler, Toledo, OH, USA. Using 25 milliliters of distilled water, 1 g of the formulation was dissolved. After dipping a pH meter probe into it, the reading was taken. Measurements of three different batches were conducted at room temperature [75].

##### Viscosity Measurement

The viscosities of the formulations were measured using the Brookfield DVE Viscosimeter (Harlow, UK). Spindle number 07 was revolved at room temperature at 100 rpm until the measured value stabilized. Measurements of three different batches were made.

##### Determination of Content Quantity in the Formulations

As a reference agent, chlorogenic acid has been chosen. One gram of the formulation was divided among five milliliters of methanol for the content quantity examination. To stop evaporation, the lid was sealed tightly and covered with parafilm. After that, it was centrifuged for 30 min at 5000 rpm. The HPLC method was used to analyze the supernatant. The SHIMADZU LC 2030C 3D Nexera-i Plus (Kyoto, Japan) apparatus was examined for HPLC analysis using a C18 column (GL Science, Tokyo, Japan −5 μm, 4.6 × 250 mm) at 30 °C and 324 nm. The injection volume was calculated to be 20 μL, and the flow rate was set at 1 mL/min. The mobile phase was produced using 0.1% formic acid/acetonitrile (85:15). Throughout the experiment, the chromatographic conditions were optimized and kept constant. In terms of system appropriateness, linearity, limit of detection (LOD) and limit of quantitation (LOQ), precision, accuracy, specificity, and selectivity, the method was partially validated in accordance with ICH criteria.

##### Spreadability and Texture Profile Analysis

The texture profiles and spreadability were assessed using a TA-XT Plus texture analyzer (Stable Micro Systems, Surrey, UK) running in TPA mode. An Eppendorf 5810R centrifuge (New York, NY, USA) was used to centrifuge 10× *g* of formulation in 50 mL falcon tubes at 4000 rpm for 10 min in order to evaluate the texture profiles. The sample was positioned 10 mm above the probe, which was lowered into the sample by 10 mm for two minutes at a steady pace of 1 mm·s^−1^ under the control of the trigger force. After retracting the probe at a speed of 0.5 mm·s^−1^, a second compression was carried out five seconds later. At a temperature of 25 °C, these analyses were conducted three times. With a test speed of 3 mm/s and a post-test speed of 10 mm/s, the spreadability test involved placing 2 g of the formulation within the female cone and moving the male cone toward it to a distance of 23 mm. Firmness (maximum positive force) and stickiness (highest negative force) were used to gauge the gel’s spreadability [32].

##### Rheology Studies of the Formulations

Each formulation’s rheological properties were measured using a Haake Mars rheometer (Thermo Fisher, Langenselbold, Germany). Tests were conducted at 25 ± 0.1 °C and 32 ± 0.1 °C using a parallel steel plate (40 mm diameter) with a 0.053 mm gap. Prior to analysis, the PKM-loaded gel and blank gel were put onto the bottom plate, with care taken to prevent sample distortion, and allowed to equilibrate for at least 1 min. Flow curves were obtained for increasing and decreasing shear velocity in the 10–1000 s^−1^ range during continuous flow conditions.

The obtained flow curves were fitted by the Herschel–Bulkley model: τ = τ0 + K˙γn, where τ (Pa) is shear stress, τ0 is the yield stress, ˙γ (s^−1^) is the shear rate, n (no dimensional) is the flow behavior index and K (Pa s^n^) is the consistency index. All given flow characteristics are the mean of at least three independent measurements.

The storage modulus (G′), loss modulus (G″), dynamic viscosity (η′), and loss tangent (tanδ) were determined by analyzing the oscillation behavior of each formulation. The linear viscoelastic area was previously determined at 25 ± 0.1 °C and 32 ± 0.1 °C. The samples were then examined using frequency scan testing in the 0.1–10 Hz spectrum, with a constant stress and a consistent gap size of 0.3 mm. To ensure data accuracy, dynamic rheological characteristics were measured in at least five different replicates for three different batches of each sample [76].

#### 4.5.2. In Vitro Release Studies

The in vitro release profile of the formulation was evaluated using the dialysis bag diffusion method. For this experiment, a precisely weighed one-gram portion of the gel containing 4% *w*/*w* PKM was placed in a regenerated cellulose dialysis membrane with a cutoff of 12−14 kDa, with tightly sealed ends. Each prepared bag was then immersed in a beaker containing 40 mL of PBS (pH 7.4). The entire setup was maintained at 32 ± 1 °C and shaken with a magnetic stirrer at 100 rpm. Samples were collected at the indicated time points (0.5, 1 h) and then every hour for 12 h, with 0.5 mL of the sample immediately replaced with an equivalent volume of fresh diffusion medium [77]. Conditions were maintained throughout the experiment. The chlorogenic acid concentration in the collected samples was measured by HPLC at 324 nm [78].

##### Kinetic Release Modeling

To understand the drug release kinetics, an evaluation of the in vitro release profile was conducted by applying the data to a range of kinetic equations. These included the zero-order, first-order, Higuchi, Hixson–Crowell and Korsmeyer–Peppas models. The degree of conformity between the empirical data and the mathematical models was assessed using the coefficient of determination (r^2^), where a greater value signified a superior fit [79].

##### Similarity Studies

In vitro release patterns of the formulations were statistically analyzed using a new mathematical method created by Moore and Flanner. This model-independent method evaluates the dissolution profiles using a similarity factor (*f*_2_) and a difference factor (*f*_1_). Comparable release profiles are indicated by F1 values up to 15 and F2 values between 50 and 100 [80]. For intra-formulation comparisons, statistical analysis was done.

Using Equations (1) and (2), the difference factor (*f*_1_) and the similarity factor (*f*_2_) were determined.

(1) f1=∑t=1nRt−Tt∑t=1nRt×100

(2) $$f_{2}=50\times log\left[{\left(1+\left(\frac{1}{n}\right)\sum {\left(R_{t}-T_{t}\right)}^{2}\right)}^{-0.5}\times 100\right]$$

R_t_ and T_t_ stand for the proportion of medication released from the reference and test formulations at time t, respectively, and n is the number of sampling time points. The dissimilarity between the two release patterns is represented by the difference factor (*f*_1_), which measures the percent divergence between them at each time point. The degree of agreement between the profiles is shown by the similarity factor (*f*_2_), which is obtained by logarithmically transforming the squared differences. These factors are interpreted using predetermined, if arbitrary, thresholds found in the literature [53].

### 4.6. In Vitro Investigation of Antioxidant Potential

CUPRAC, TOAC, FRAP, and DPPH radical scavenging tests were used to measure antioxidant activity. By measuring a change in absorbance at 517 nm following a 30 min incubation period in the dark, the DPPH assay calculates the reduction in DPPH radicals. The FRAP assay measures absorbance at 593 nm following a 30 min incubation period at 37 °C. The reduction of Fe(III) to Fe(II) produces a colorful complex. Using ascorbic acid as the reference, the CUPRAC and TOAC assays evaluated the samples’ capacity to reduce Cupric and Mo (VI) ions, respectively. Every experiment was run in triplicate, and the mean values ± standard deviation were used to express the results [81].

### 4.7. Skin Enzyme Inhibition Assays

#### 4.7.1. Anti-Collagenase Activity

In total, 400 mM NaCl and 10 mM CaCl_2_ were added to a 50 mM Tricine buffer solution at pH 7.5. This buffer was used to dissolve the Clostridium histolyticum collagenase enzyme, resulting in an initial concentration of 0.8 units/mL. The substrate, *N*-[3-(2-furyl)-acryloyl]-Leu-Gly-Pro-Ala (FALGPA), was dissolved in the Tricine buffer at a concentration of 2 mM. Before the substrate was added to initiate the reaction, the extracts were incubated with the collagenase enzyme in the buffer for fifteen minutes. The final reaction mixture contained 25 μg of the test extract, 0.8 mM FALGPA, and 0.1 unit ChC in a total volume of 150 μL. Absorbance was measured right after the substrate was added in negative controls, which solely employed the Tricine buffer. As a positive control, EGCG was employed [82].

#### 4.7.2. Anti-Elastase Activity

A 0.2 mM tris-HCl buffer (pH 8.0) and a stock solution of elastase enzyme (3.33 mg/mL) made in sterile water were used to assess the extracts’ antielastase activity. *N*-succinyl-Ala-Ala-p-nitroanilide (AAAPVN), the substrate, was dissolved in the buffer at a 1.6 mM concentration. Before the substrate was added, the plant extracts were preincubated with the enzyme for fifteen minutes. Methanol served as the negative control and epigallocatechin gallate (EGCG) as the positive control. A Thermo Scientific Multiskan SkyHigh Microplate Spectrophotometer (Thermo Fisher, Langenselbold, Germany) was used to detect absorbance changes after the mixture was incubated for an additional 15 min after the addition of the substrate [83].

#### 4.7.3. Anti-Hyaluronidase Activity

A 10% concentration of cetylpyridinium chloride solution and a 50 mM (pH: 7) Tris-HCL buffer solution were made (*w*/*v*) in order to assess the extracts’ antihyaluronidase activity. Distilled water was used to dissolve 800 U/mL of hyaluronic acid enzyme and 0.4 mg/mL of hyaluronic acid substrate. Briefly, 10 μL of substrate,10 μL of enzyme, 10 μL of plant extract, and 70 μL of Tris-HCL buffer solution make up the final volume of 110 μL. First, 10 μL of plant extract, 10 μL of enzyme, and 70 μL of buffer solution were incubated for an hour at 37 °C. After that, 10 μL of cetylpyridinium chloride was added, and the mixture was incubated at 37 °C for an additional hour. After adding 10 μL of substrate, absorbance readings were measured. Distilled water was used to create negative controls. The reference tannic acid concentration was 1.1 mg/mL. At 415 nm, absorbances were measured [74].

#### 4.7.4. Antityrosinase Activity

A modified method based on the work of Er-soy et al. was used to measure in vitro antityrosinase activity. L-DOPA was utilized as the substrate in a 100 mM phosphate buffer with a pH of 6.8. First, 20 μL of a 1 mg/mL plant extract, 20 μL of tyrosinase enzyme, and 100 μL of phosphate buffer were incubated for ten minutes at 37 °C. After adding 20 μL of 3 mM DOPA, the mixture was once more incubated for 30 min at 37 °C. Kojic acid was utilized as the reference standard when measuring the absorbance at 492 nm [84].

### 4.8. Statistical Analysis

The experiments presented in this article were repeated at least three times in independent time spans. All experiments were performed with GraphPad Prism 8.0 software.

## Figures and Tables

**Table 1:** LC-MS/MS results of PKM extract.

	Analytes	RT ^a^	M.I. (*m*/*z*) ^b^	F.I. (*m*/*z*) ^c^	Ion. Mode	Quantification (μg/g)
1	Quinic acid	3.0	190.8	93.0	-	85.396
2	Fumaric aid	3.9	115.2	40.9	-	0.7
3	Equisetic acid	4.0	172.8	129.0	-	-----
4	Gallic acid	4.4	168.8	79.0	-	0.127
5	Epigallocatechin	6.7	304.8	219.0	-	-----
6	Protocatechuic acid	6.8	152.8	108.0	-	1.757
7	Catechin	7.4	288.8	203.1	-	-----
8	Gentisic acid	8.3	152.8	109.0	-	-----
9	Chlorogenic acid	8.4	353.0	85.0	-	20.379
10	Protocatechuic aldehyde	8.5	137.2	92.0	-	0.471
11	Tannic acid	9.2	182.8	78.0	-	0.019
12	Epigallocatechin gallate	9.4	457.0	305.1	-	-----
13	Cynarin	9.8	515.0	191.0	-	-----
14	4-OH Benzoic acid	10.5	137.2	65.0	-	0.232
15	Epicatechin	11.6	289.0	203.0	-	-----
16	Vanillic acid	11.8	166.8	108.0	-	-----
17	Caffeic acid	12.1	179.0	134.0	-	0.853
18	Syringic acid	12.6	196.8	166.9	-	-----
19	Vanillin	13.9	153.1	125.0	+	0.322
20	Syringic aldehyde	14.6	181.0	151.1	-	0.059
21	Daidzin	15.2	417.1	199.0	+	-----
22	Epicatechin gallate	15.5	441.0	289.0	-	-----
23	Piceid	17.2	391.0	135/106.9	+	-----
24	p-Coumaric acid	17.8	163.0	93.0	-	0.16
25	Ferulic acid-D3-IS^h^	18.8	196.2	152.1	-	N.A.
26	Ferulic acid	18.8	192.8	149.0	-	-----
27	Sinapic acid	18.9	222.8	193.0	-	-----
28	Coumarin	20.9	146.9	103.1	+	-----
29	Salicylic acid	21.8	137.2	65.0	-	1.851
30	Luteolin 7-*O*-glucoside	23.7	447.0	284.0	-	5.373
31	Miquelianin	24.1	477.0	150.9	-	-----
32	Rutin-D3-IS	25.5	612.2	304.1	-	N.A.
33	Rutin	25.6	608.9	301.0	-	0.212
34	Isoquercetin	25.6	463.0	271.0	-	2.729
35	Hesperidin	25.8	611.2	449.0	-	0.068
36	o-Coumaric acid	26.1	162.8	93.0	-	-----
37	Genistin	26.3	431.0	239.0	-	-----
38	Rosmarinic acid	26.6	359.0	197.0	-	-----
39	Ellagic acid	27.6	301.0	284.0	-	-----
40	Apigenin 7-glucoside	28.2	431.0	269.0	-	2.397
41	Quercitrin	29.8	447.0	301.0	-	-----
42	Astragalin	30.4	447.0	255.0	-	0.249
43	Nicotiflorin	30.6	592.9	255.0/284.0	-	-----
44	Fisetin	30.6	285.0	163.0	-	-----
45	Daidzein	34.0	253.0	223.0	-	-----
46	Quercetin-D3-IS	35.6	304.0	275.9	-	N.A.
47	Quercetin	35.7	301.0	272.9	-	0.044
48	Naringenin	35.9	270.9	119.0	-	2.062
49	Hesperetin	36.7	301.0	136.0/286.0	-	-----
50	Luteolin	36.7	284.8	151.0/175.0	-	0.966
51	Genistein	36.9	269.0	135.0	-	0.009
52	Kaempferol	37.9	285.0	239.0	-	0.039
53	Apigenin	38.2	268.8	151.0/149.0	-	0.444
54	Amentoflavone	39.7	537.0	417.0	-	0.002
55	Chrysin	40.5	252.8	145.0/119.0	-	0.004
56	Acacetin	40.7	283.0	239.0	-	0.545

^a^: retention time, ^b^: MI (*m*/*z*): molecular ions of the standard analytes (*m*/*z* ratio), ^c^: FI (*m*/*z*): fragment ions N.A.: not applicable, N.D.: not detected, (-): Neg, (+): Poz.

**Table 2:** Quantification of chlorogenic acid by HPTLC.

Compound	PKM	PKH	PKC	PKE	PKB	PKW
Chlorogenic acid	56.88 ± 0.89	40.14 ± 1.72	14.27 ± 0.68	205 ± 16.73	125.97 ± 7.09	48.16 ± 0.57

Quantitative data of chlorogenic acid expressed as μg/g in all fractions using HPTLC analysis. PKM: methanolic extract of PK, PKH: hexane fraction of PK, PKC: chloroform fraction of PK, PKE: ethyl acetate fraction of PK, PKB: *n*-butanol fraction of PK, PKW: remaining water fraction of PK.

**Table 3:** Characterization results of blank and loaded formulations.

Formulation/Characterization	F	PKF
pH	5.40 ± 0.10	6.03 ± 0.01
Viscosity (P)	24.023 ± 0.228	19.027 ± 0.206
Extract Content (%)	-	101.069 ± 5.375

F: blank gel, PKF: emulgel loaded with methanolic extract of PK (PKM).

**Table 4:** Texture characteristics results of the analysis for blank and methanol extract-loaded formulations, encompassing hardness, adhesiveness, elasticity, and cohesiveness (Mean ± SD).

Formulation	Hardness (g) ± SS	Adhesiveness (g.s) ± SS	Elasticity ± SS	Cohesiveness ± SS
F	32.512 ± 0.195	−90.678 ± 0.391	0.235 ± 0.053	0.855 ± 0.139
PKF	16.275 ± 0.481	−43.886 ± 1.448	0.180 ± 0.018	0.873 ± 0.092

**Table 5:** Results of spreadability for blank formulations and those containing methanol extract, encompassing firmness, work of shear, stickiness, and work of adhesion (Mean ± SD).

Formulation	Firmness (g)	Work of Shear (g.s)	Stickiness (g)	Work of Adhesion (g.s)
F	1591.67 ± 6.01	1657.73 ± 32.20	−1287.72 ± 14.37	−214.75 ± 12.33
PKF	1174.39 ± 38.54	1102.00 ± 72.16	−978.00 ± 1.30	−200.73 ± 8.11

**Table 6:** Effect of temperature on the loss tangent tan (δ) and on the dynamic viscosity (η’) of the formulations at three representative frequencies.

Formulation	Temperature (°C)	tan (δ) Values at Different Oscillation Frequencies		
		0.1 Hz	1 Hz	10 Hz
F	25	0.09 ± 0.01	0.06 ± 0.01	0.06 ± 0.01
	32	0.07 ± 0.00	0.05 ± 0.00	0.07 ± 0.01
PKF	25	0.04 ± 0.02	0.04 ± 0.01	0.06 ± 0.01
	32	0.14 ± 0.05	0.06 ± 0.01	0.07 ± 0.01
Formulation	Temperature (°C)	η* (Pas) at different oscillation frequencies		
		0.1 Hz	1 Hz	10 Hz
F	25	103.72 ± 35.64	8.71 ± 2.07	0.92 ± 0.16
	32	93.31 ± 1.56	7.36 ± 0.21	1.10 ± 0.01
PKF	25	35.36 ± 14.99	3.88 ± 0.56	0.50 ± 0.10
	32	117.09 ± 39.95	5.75 ± 1.98	0.78 ± 0.23

**Table 7:** In vitro release kinetics results of PKS and PKF.

Formulations	PKS			PKF		
Models	r^2^	n	k	r^2^	m	n
Zero-order	0.922		52.105	0.961		59.462
First-order	0.849		3.972	0.938		4.116
Hixson–Crowell	0.876		5.559	0.947		6.21
Higuchi	0.989		23.425	N/A *		N/A
Korsmeyer–Peppas	0.996	0.2802	1.717	0.977	0.238	1.876

* N/A: Not Available.

**Table 8:** The calculated difference (*f*_1_) and similarity (*f*_2_) factor for PKS and PKF.

Method of Release	Reference Formulation	Experimental Formulation	*f*_1_	*f*_2_
Dialysis bag	PKS	PKF	23	33
	PKF	PKS	30	33

**Table 9:** In vitro tests for the antioxidant potential of different extracts of PK and PKF.

	DPPH ^1^	FRAP ^2^	CUPRAC ^3^	TOAC ^3^
PKM *	1849 ± 58 ^a^	0.87 ± 0.04 ^a^	130.32 ± 10.31 ^a^	159.33 ± 11.70 ^ad^
PKH *	1030 ± 35 ^b^	0.42 ± 0.08 ^b^	65.05 ± 4.85 ^b^	133.49 ± 12.93 ^a^
PKC *	1030 ± 35 ^b^	0.51 ± 0.06 ^b^	92.13 ± 5.94 ^bc^	162.93 ± 26.68 ^ad^
PKE *	2515 ± 37 ^d^	2.27 ± 0.06 ^c^	326.53 ± 19.76 ^d^	259.53 ± 6.04 ^b^
PKB *	2628 ± 28 ^e^	1.86 ± 0.03 ^d^	300.73 ± 9.31 ^d^	221.56 ± 7.03 ^c^
PKW *	1750 ± 33 ^a^	0.73 ± 0.03 ^a^	101.34 ± 8.55 ^c^	132.80 ± 1.11 ^a^
PKF	989 ± 35 ^b^	0.76 ± 0.09 ^a^	144.65 ± 4.04 ^a^	181.17 ± 5.51 ^d^

^1^ Results were expressed as the mean of triplicates ± standard deviation (S.D.) and as mg butylated hydroxyl toluene equivalents (BHTE) in 1 g sample. ^2^ Results were expressed as the mean of triplicates ± standard deviation (S.D.) and as mM FeSO_4_ equivalents in 1 g sample. ^3^ Results were expressed as the mean of triplicates ± standard deviation (S.D.) and as mg ascorbic acid equivalents (AAE) in 1 g sample. * PKM: methanolic extract of PK, PKH: hexane fraction of PK, PKC: chloroform fraction of PK, PKE: ethyl acetate fraction of PK, PKB: n-butanol fraction of PK, PKW: remaining water fraction of PKW, PKF: Emulgel formulation loaded with methanol extract of PK. Different letters in the same row indicate significance (*p* < 0.05).

## Data Availability

The datasets used and/or analyzed during the current study are available from the corresponding author on reasonable request.

## Abbreviations

AAAPVN	*N*-succinyl-Ala-Ala-p-nitroanilide
CUPRAC	Cupric reducing antioxidant capacity
ECM	Extracellular matrix
EGCG	Epigallocatechin gallate
DPPH	2,2-difenil-1-pikrilhidrazil
FALGPA	*N*-[3-(2-furyl)-acryloyl]-Leu-Gly-Pro-Ala
FRAP	Ferric Reducing Antioxidant Power
GC-MS	Gas chromatography-mass spectrometry
HLB	Hydrophilic–lipophilic balance
HPTLC	High-Performance Thin Layer Chromatography
LC-MS/MS	Liquid Chromatography Tandem-mass Spectrometry
LOD	Limit of detection
LOQ	Limit of quantitation
PK	*Phlomis kurdica*
PKM	Methanolic extract of *Phlomis kurdica*
ROS	Reactive oxygen species
TOAC	Total Antioxidant Capacity
TPA	Texture profile analysis
